# The value of aspartate aminotransferase and alanine aminotransferase in cardiovascular disease risk assessment

**DOI:** 10.1136/openhrt-2015-000272

**Published:** 2015-08-21

**Authors:** Stephen F Weng, Joe Kai, Indra Neil Guha, Nadeem Qureshi

**Affiliations:** 1 Division of Primary Care, NIHR School of Primary Care Research, University of Nottingham, Nottingham, UK; 2 NIHR Biomedical Research Unit in Gastrointestinal and Liver Diseases at Nottingham University Hospitals NHS Trust and the University of Nottingham, Nottingham, UK

## Abstract

**Objective:**

Aspartate aminotransferase to alanine aminotransferase (AST/ALT) ratio, reflecting liver disease severity, has been associated with increased risk of cardiovascular disease (CVD). The aim of this study was to evaluate whether the AST/ALT ratio improves established risk prediction tools in a primary care population.

**Methods:**

Data were analysed from a prospective cohort of 29 316 UK primary care patients, aged 25–84 years with no history of CVD at baseline. Cox proportional hazards regression was used to derive 10-year multivariate risk models for the first occurrence of CVD based on two established risk prediction tools (Framingham and QRISK2), with and without including the AST/ALT ratio. Overall, model performance was assessed by discriminatory accuracy (AUC c*-*statistic).

**Results:**

During a total follow-up of 120 462 person-years, 782 patients (59% men) experienced their first CVD event. Multivariate models showed that elevated AST/ALT ratios were significantly associated with CVD in men (Framingham: HR 1.37, 95% CI 1.05 to 1.79; QRISK2: HR 1.40, 95% CI 1.04 to 1.89) but not in women (Framingham: HR 1.06, 95% CI 0.78 to 1.43; QRISK2: HR 0.97, 95% CI 0.70 to 1.35). Including the AST/ALT ratio with all Framingham risk factors (AUC c*-*statistic: 0.72, 95% CI 0.71 to 0.74) or QRISK2 risk factors (AUC c*-*statistic: 0.73, 95% CI 0.71 to 0.74) resulted in no change in discrimination from the established risk prediction tools. Limiting analysis to those individuals with raised ALT showed that discrimination could improve by 5% and 4% with Framingham and QRISK2 risk factors, respectively.

**Conclusions:**

Elevated AST/ALT ratio is significantly associated with increased risk of developing CVD in men but not women. However, the ratio does not confer any additional benefits over established CVD risk prediction tools in the general population, but may have clinical utility in certain subgroups.

**Key messages**

**What is already known about this subject?**

- The aspartate aminotransferase to alanine aminotransferase (AST/ALT) ratio, reflecting liver disease severity, has been associated with increased risk of cardiovascular disease (CVD) in patients with liver disease. It is unknown whether these simple, routinely collected biomarkers, can improve CVD prediction in a general primary care population.

**What does this study add?**

- By following-up a large UK cohort of primary care patients over 10 years, we have found that the AST/ALT ratio is significantly associated with increased risk of CVD in men but not women. However, the ratio does not confer any additional benefits in predictive accuracy for predicting CVD when included in standard primary care-based risk prediction tools.

**How might this impact on clinical practice?**

- Our analysis suggests the AST/ALT ratio should not be included in current CVD risk prediction tools for the general primary care population. However, in the era of stratified medicine, those with raised AST/ALT ratio may represent a higher risk subgroup that could benefit from closer monitoring, particularly when the ALT is raised.

## Introduction

Cardiovascular disease (CVD) continues to be the leading cause of death in the developed world. In the UK, there are an estimated 180 000 deaths per year from CVD.^1^ The UK National Institute for Health and Care Excellence (NICE) lipid modification guidelines recommend the use of risk prediction tools, to identify individuals at increased risk of CVD.^2^ In the UK, QRISK2^3^ is recommended while internationally, the Framingham tool^4^ is adopted. These tools calculate CVD risk using recognised risk factors, such as hypertension, cholesterol levels, age, smoking and family history of premature CVD.

Although most CVD events can be attributed to the aforementioned major risk factors embedded within routinely-used risk prediction tools, a significant proportion of the population will experience an event in the absence of traditional risk factors. For example, up to 50% of all myocardial infarctions (MI) and strokes occur in individuals with low-density lipoprotein cholesterol levels below the recommended thresholds for lipid modification.^5^ In the USA, the National Cholesterol Education Program (NCEP) Adult Treatment Panel (ATP) III now recommends that to effectively reduce the CVD risk of such patients requires consideration of ‘emerging risk factors’.^6^

Liver function enzymes aspartate aminotransferase (AST) and alanine aminotransferase (ALT) are examples of emerging biomarkers for CVD risk. The AST to ALT has been shown to reflect disease severity in a number of chronic liver diseases, including alcoholic and non-alcoholic liver disease,^7^ autoimmune liver disease^8^ and hepatitis C.^9^ Previous studies^10–14^ have demonstrated that CVD is the leading cause of death in non-alcoholic fatty liver disease (NAFLD), with higher rates coinciding with higher liver-related mortality over follow-up periods from 10 to 20 years.

The detection and identification of liver disease in the community is limited. Liver disease is asymptomatic until the final stages of cirrhosis. However, using the enzymes in a simple ratio (AST/ALT) or as a component of panel marker tests has been shown to have diagnostic accuracy for significant liver disease.^15^ ^16^ In addition, these surrogates of liver injury improve the prediction of future clinical events including cardiovascular outcomes in patients with liver disease.^17^ ^18^ However, it is still unknown whether the AST/ALT ratio can improve prediction for cardiovascular outcomes in a general primary care population. These simple liver markers are routinely available in primary care, offering potential utility in primary care-based risk prediction models. Therefore, the aim of this study was to evaluate whether the inclusion of AST/ALT ratio improves standard 10-year risk prediction models for CVD in a primary care population.

## Methods

### Data sources

Data were obtained from the Clinical Practice Research Datalink (CPRD), a cohort of patients with prospectively collected data, derived from anonymised electronic medical records of more than 12 million patients from 681 UK general practices. Approximately 8% of the UK population is included and the database is broadly representative of the UK population.^19^ Additionally, 55% of the CPRD patients are also linked to secondary care across UK, through Hospital Episode Statistics (HES) database, which document hospital admissions, diagnoses (ICD-10), and treatment received.

Data undergo quality checks and practices are designated as meeting internal quality criteria for research purposes and over 550 peer-reviewed studies have been published from the databases.^19^ Ethical approval was granted by the CPRD Independent Scientific Advisory Committee (protocol 14_080).

### Study population

The study population consisted of registered patients at a CPRD General Practice aged between 25 and 84 years (covering the age range of the CVD risk prediction tool) at baseline, with complete AST and ALT measurements recorded within 2 days of one another prior to the baseline date, and who had complete data recorded on seven recognised core risk factors (sex, age, smoking status, systolic blood pressure, blood pressure treatment, total cholesterol (TC) and high-density lipoprotein (HDL) cholesterol) prior to the baseline date. The baseline date was specified as 1 January 2004, which enabled patients to have a 10-year follow-up for estimating 10-year CVD risk. The end of the study period was the most recent date for which data were available (1 January 2014). Patients were excluded if they had a pre-existing history of CVD or inherited lipid disorders prior to baseline, were on lipid lowering drugs prior to baseline, or outside the specified age range at baseline.

### Risk factors

The core risk factors for predicting CVD were derived from the variables included in the 10-year Framingham and QRISK2 risk algorithms (table 1). The core factors encompass all eight variables in the Framingham risk algorithm (age, gender, systolic blood pressure, blood pressure treatment, TC, HDL cholesterol, diabetes and smoking). The QRISK2 risk algorithms consists of 14 variables, including all risk factors present in the Framingham, with the addition of body mass index (BMI), family history of premature coronary heart disease (CHD), chronic kidney disease, atrial fibrillation, rheumatoid arthritis, Townsend deprivation score and ethnicity. Blood pressure treatment was classified as a diagnosis of hypertension and at least one current prescription of an antihypertensive agent. The AST/ALT ratio was included as a novel marker to these original risk algorithms. We used these risk factors to derive new sets of regression functions which were calibrated for the CPRD study population, a common approach in risk prediction modelling.^20^

**Table 1:** Risk factors included in the standard 10-year Framingham and QRISK2 cardiovascular risk prediction algorithms

Risk factors	Framingham risk factors	QRISK2 risk factors
Age (years)	✓	✓
Gender (male/female)	✓	✓
Systolic blood pressure (mm Hg)	✓	✓
Blood pressure treatment (yes/no)	✓	
Total cholesterol (mmol/L)	✓	
HDL cholesterol (mmol/L)	✓	✓
Total cholesterol/HDL ratio		✓
Body mass index (kg/m^2^)		✓
Diabetes (yes/no)	✓	✓
Family history of CHD <60 years (yes/no)		✓
Chronic kidney disease (yes/no)		✓
Atrial fibrillation (yes/no)		✓
Rheumatoid arthritis (yes/no)		✓
Smoking (yes/no)	✓	✓
Ethnicity (Caucasian/non-Caucasian)		✓
Townsend score (quintiles)		✓
AST/ALT ratio*		

*Novel marker.

ALT, alanine transaminase; AST, aspartate transaminase; CHD, coronary heart disease; HDL, high-density lipoprotein.

### Outcome

The outcome of interest for this analysis was a diagnosis of the first CVD event (fatal or non-fatal) in a patient's electronic medical records during the follow-up period of 10 years after the baseline date (1 January 2014). The medical diagnosis of CVD is coded electronically in National Health Service (NHS) primary care electronic health records as Read Codes (full details in online supplementary materials). Further, for those patients who were linked to secondary care (HES) during the specified study period (55% of sample), we utilised ICD-10 codes (I20-25 for coronary heart disease, I60–69 for cerebrovascular disease) to validate the CVD outcome recorded in primary care. If the secondary care CVD event recorded occurred prior to the primary care CVD event, then the date of the first event was determined by the secondary care record and vice versa. Patients who died due to other causes or transferred before the end of follow-up were censored from the analysis.

### Statistical analysis

As the Framingham risk scores could be calculated from the core risk factors, there were no missing values. However, to calculate QRISK2 risk scores, there were missing values for additional variables of BMI, ethnicity and Townsend deprivation score. Standard procedure of multiple imputation was used to deal when missing values where identified in QRISK2 variables with 10 copies of the missing data imputed. This approach follows the same imputation procedures utilised in the original QRISK2 algorithm.^3^ A descriptive analysis was performed, reporting number (%), mean (SD) and median (IQR) for categorical, normal continuous and non-normal continuous variables, respectively.

Multivariate prediction models were derived from Cox proportional hazards regression, using the original risk factors included in Framingham and QRISK2, which gave the probability of the first onset of the CVD outcome. All continuous variables were log-transformed to preserve normality in the multivariate models. In addition to patients being censored as a result of loss to follow-up (death or transfer), patients who were prescribed lipid-lowering drug treatment, during follow-up, were also censored as these patients would likely have significantly altered their risk during the follow-up period. We then incorporated the AST/ALT ratio into both multivariate models to derive a new set of prediction models. These functions were then applied to each patient to calculate a new patient risk score with the addition of the AST/ALT ratio. The process of developing the linear prediction model from Cox regression to calculate patient 10-year CVD risk is described in detail in online supplementary materials. All underlying assumptions were investigated for the Cox models including proportional hazards, influential and outlying observations.

The performance of the multivariate prediction models was assessed by the area under the receiver operating characteristics curves (AUC), estimated as Harrell's c*-*statistic, with higher values representing better discrimination (*ability to distinguish between a case and a non-case)*. To generate CIs for the c*-*statistic, a jack-knife procedure^21^ was used to bootstrap SEs. All analyses were performed using STATA V.13 MP4.

## Results

### Characteristics of the study population

There were 30 929 patients at baseline (1 January 2004) with a documented AST/ALT ratio and complete data for eight core risk factors (age, gender, systolic blood pressure, blood pressure treatment, TC, HDL cholesterol, diabetes and smoking). From this starting sample, 1613 patients were removed due to outlying observations for cholesterol and blood pressure, or data entry errors (non-numerical entries). The outlying observations were determined by reference ranges, plus/or minus 10%, determined from data extracted from the Health Survey for England^22^ (ranges shown in table 2). This process is a validated approach^23^ in removing outliers for UK general practice database studies using the Health Survey for England.

**Table 2:** Reference ranges derived from data extracted from the 2012 health survey for England

Variable	Reference ranges	
	Men	Women
Total cholesterol (mmol/L)	2.1–10.0	2.3–11.9
HDL cholesterol (mmol/L)	0.4–3.7	0.5–5.0
Systolic blood pressure (mm Hg)	70–220	59–221
Body mass index (kg/m^2^)	7.5–65.3	10.3–61.8

HDL, high-density lipoprotein.

The remaining 29 316 patients (52% female) were included for the analysis as the study cohort in which patients were followed-up for a total of 120 462 person-years (men: 55 606 person-years; women: 64 856 person-years). During the follow-up period, there were 1241 patients (4.2%) who died due to other causes and 2701 (9.2%) patients who were lost to follow-up due to transferring from the practice. Additionally, 2085 patients (7.1%) were censored due to starting statin treatment during the follow-up period. The remaining patients were alive and at the practice at the end of follow-up. There were a total of 782 patients (461 men, 321 women) who had a CVD event, with a slightly higher proportion occurring in men than in women. The median age of the cohort for men was 58 years (IQR: 49–68) and for women was 61 years (IQR: 52–71). The median AST/ALT differed between men and women (0.8 IQR: 0.7–1.1 for men; 1.0 IQR: 0.8–1.3 for women). Median TC and HDL cholesterol were lower for men than in women while median systolic blood pressure and BMI were similar between men and women. Missing data also occurred in three variables used in the QRISK2 algorithm. BMI was not recorded in 72% of men and 73% of women, ethnicity missing for 51% of men and 48% of women, and Townsend score was missing for 36% of men and 38% of women. Further details of all descriptive characteristics are presented in table 3.

**Table 3:** Characteristics of patients aged 25–84 years in the CPRD study cohort stratified by gender with a 10-year follow-up

Characteristics	N (%) of men	N (%) of women
Patients	14 175 (0.48)	15 141 (0.52)
Total person-years	55 606	64 856
CVD event	461 (0.03)	321 (0.02)
Median age (IQR)	58 (49–68)	61 (52–71)
Median total cholesterol in mmol/L (IQR)	5.3 (4.6–6.0)	5.5 (4.8–6.3)
Median HDL cholesterol in mmol/L (IQR)	1.3 (1.1–1.5)	1.5 (1.3–1.8)
Median total cholesterol/HDL ratio (IQR)	4.2 (3.4–5.0)	3.6 (2.9–4.4)
Median systolic blood pressure in mmol/L (IQR)	140 (128–150)	140 (126–150)
Median body mass index in kg/m^2^ (IQR)*	26.3 (23.3–30.0)	26.4 (22.5–31.1)
Body mass index missing	12 554 (0.89)	13 339 (0.88)
Median AST/ALT ratio (IQR)	0.8 (0.7–1.1)	1.0 (0.8–1.3)
On blood pressure treatment	3070 (0.22)	3559 (0.24)
Smoking	3222 (0.23)	3066 (0.20)
Diabetes	2678 (0.19)	2305 (0.15)
Chronic kidney disease	124 (0.009)	84 (0.006)
Atrial fibrillation	386 (0.03)	315 (0.02)
Rheumatoid arthritis	118 (0.008)	296 (0.02)
Family history of premature CHD <60 years	719 (0.05)	771 (0.05)
Ethnicity*		
Caucasian	5522 (0.39)	6493 (0.43)
Non-Caucasian	1368 (0.10)	1382 (0.09)
Missing	7285 (0.51)	7266 (0.48)
Townsend quintiles		
1st—least deprived	2739 (0.19)	2499 (0.17)
2nd	2085 (0.15)	2046 (0.14)
3rd	1679 (0.12)	1875 (0.12)
4th	1622 (0.11)	1837 (0.12)
5th—most deprived	940 (0.06)	1149 (0.08)
Missing	5120 (0.36)	5735 (0.38)

*Variable contains missing values.

Values are number and proportions unless otherwise stated.

ALT, alanine transaminase; AST, aspartate transaminase; CVD, cardiovascular disease; CHD, coronary heart disease; CRPD, Clinical Practice Research Datalink; HDL, high-density lipoprotein.

### Multivariate hazard models

#### Risk factors in Framingham risk prediction model

Two multivariate hazard regression models were developed for both the Framingham and QRISK2 risk factors. The Framingham risk factor model shown in table 4 includes all original Framingham risk factors with the addition of the AST/ALT ratio.

**Table 4:** Multivariate hazard model showing adjusted HRs and corresponding 95% CI for calibrated Framingham risk factors including AST/ALT ratio

	Adjusted HR (95% CI)	
Risk factors	Men	Women
Age (years)*	53.33 (30.64 to 92.83)†	158.65 (76.38 to 329.55)†
Total cholesterol (mmol/L)*	1.05 (0.63 to 1.77)	1.10 (0.58 to 2.09)
HDL cholesterol (mmol/L)*	0.53 (0.37 to 0.76)†	0.43 (0.28 to 0.66)†
Systolic blood pressure if treated (mm Hg)*	2.07 (1.02 to 4.43)†	2.62 (1.06 to 6.52)†
Systolic blood pressure if no treatment (mm Hg)*	2.05 (1.01 to 4.36)†	2.67 (1.08 to 6.58)†
Smoking		
No	Ref	Ref
Yes	1.48 (1.20 to 1.83)†	2.09 (1.61 to 2.70)†
Diabetes		
No	Ref	Ref
Yes	1.35 (1.02 to 1.77)†	1.69 (1.16 to 2.46)†
AST/ALT ratio*	1.37 (1.05 to 1.79)†	1.06 (0.78 to 1.43)

*Variable log transformed.

†p<0.05.

ALT, alanine transaminase; AST, aspartate transaminase; HDL, high-density lipoprotein.

Age was the most dominant predictor of CVD for men and women. Systolic blood pressure, smoking, diabetes significantly increased risk of CVD while higher HDL cholesterol significantly reduced risk of CVD for men and women. TC levels were not found to be significantly associated with CVD. Higher AST/ALT ratio significantly increased risk of CVD in men but not women.

#### Risk factors in QRISK2 risk prediction model

The QRISK2 risk factor model shown in table 5 includes all original QRISK2 risk factors with the addition of the AST/ALT ratio.

**Table 5:** Multivariate hazard model showing adjusted HRs and 95% CI for calibrated QRISK2 risk factors including AST/ALT ratio

	Adjusted HR (95% CI)	
Risk factors	Men	Women
Age (years)*	63.80 (34.65–117.48)†	112.76 (50.92–249.70)†
Total cholesterol/HDL cholesterol ratio*	1.33 (1.01–1.92)†	2.05 (1.33–3.16)†
Systolic blood pressure (mm Hg)*	1.84 (1.04–3.96)†	2.80 (1.12–7.02)†
Body mass index (kg/m^2^)*	2.84 (0.84–9.60)	0.62 (0.16–2.33)
Blood pressure treatment		
No	Ref	Ref
Yes	0.89 (0.71–1.12)	1.08 (0.83–1.39)
Smoking		
No	Ref	Ref
Yes	1.51 (1.21–1.89)†	1.97 (1.50–2.59)†
Diabetes		
No	Ref	Ref
Yes	1.36 (1.03–1.79)†	1.84 (1.27–2.66)†
Chronic kidney disease		
No	Ref	Ref
Yes	1.97 (1.02–3.99)†	2.85 (1.05–7.74)†
Atrial fibrillation		
No	Ref	Ref
Yes	1.91 (1.32–2.76)†	2.32 (1.49–3.62)†
Rheumatoid arthritis		
No	Ref	Ref
Yes	1.50 (0.74–3.02)	1.47 (0.80–2.69)
Townsend fifths		
1st—least deprived	Ref	Ref
2nd	1.12 (0.86–1.46)	0.96 (0.69–1.32)
3rd	1.37 (1.04–1.79)†	0.86 (0.61–1.21)
4th	1.33 (1.02–1.74)†	1.16 (0.84–1.60)
5th—most deprived	1.12 (0.77–1.62)	1.28 (0.88–1.86)
Ethnicity		
Caucasian	Ref	Ref
Non-Caucasian	0.75 (0.56–0.99)	0.86 (0.61–1.21)
AST/ALT Ratio*	1.40 (1.04–1.89)†	0.97 (0.70–1.35)

*Variable log transformed.

†p<0.05.

ALT, alanine transaminase; AST, aspartate transaminase; HDL, high-density lipoprotein.

Similar to the Framingham risks factors model, the effect sizes of the core risk factors in the QRISK2 was similar. Again, age was the most dominant predictor of CVD for men and women. Systolic blood pressure, TC to HDL cholesterol ratio, smoking and diabetes were significantly associated with increased risk of CVD for men and women. Additional risk factors of chronic kidney disease, and atrial fibrillation were also associated with an increased risk of CVD for men and women. Townsend index showed that living in more deprived areas generally increased risk of CVD but this relationship was not significant in women. However, BMI, blood pressure treatment, rheumatoid arthritis and ethnicity were not found to be associated with CVD. The AST/ALT ratio was significantly associated with an increase in risk of CVD in men but not women.

### Discrimination analysis

Receiver operating curves were derived for several models in table 6.

**Table 6:** Discrimination of the prediction models derived from multivariate hazard models

Models	AUC c-statistic	SE*	95% CI
AST/ALT excluded			
Model 1a: Age+gender	0.68	0.009	0.66 to 0.70
Model 2a: Framingham risk factors	0.72	0.009	0.70 to 0.74
Model 3a: QRISK2 risk factors	0.73	0.008	0.71 to 0.74
AST/ALT ratio included			
Model 1b: Age+gender+AST/ALT ratio	0.69	0.009	0.67 to 0.71
Model 2b: Framingham risk factors+AST/ALT ratio	0.72	0.009	0.71 to 0.74
Model 3b: QRISK2 risk factors+AST/ALT ratio	0.73	0.008	0.71 to 0.74
Comparator models			
Model 4: Age+gender+systolic blood pressure	0.69	0.009	0.67 to 0.71
Model 5: Age+gender+TC/HDL ratio	0.69	0.009	0.67 to 0.71

Higher area under receiver operating curve (AUC c-statistic) shows better model performance.

SE estimated by jack-knife procedure.

ALT, alanine transaminase; AST, aspartate transaminase; HDL, high-density lipoprotein; TC, total cholesterol.

**Figure 1:**
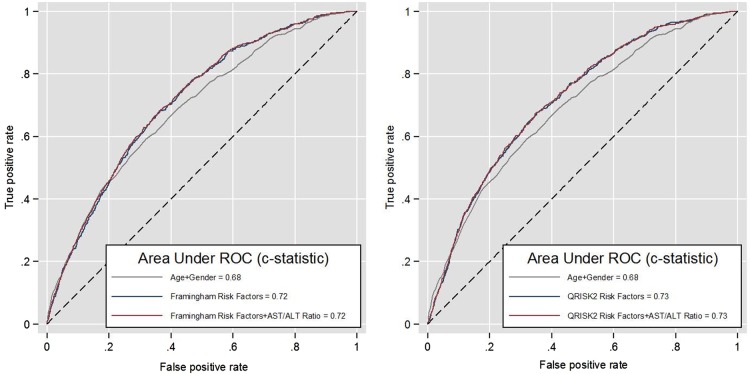
Receiver operating curves (ROC) of the Framingham and QRISK2 risk factor models for predicting 10-year risk of cardiovascular disease with and without the inclusion of the AST/ALT ratio. ALT, alanine transaminase; AST, aspartate transaminase.

The first set of models excludes the AST/ALT ratio. A simple age and gender-adjusted model (Model 1a) resulted in an AUC c*-*statistic of 0.68 (95% CI 0.66 to 0.70). The addition of all Framingham risk factors (Model 2a) improved discrimination to 0.72 (95% CI 0.70 to 0.74) while the addition of QRISK2 risk factors (Model 3a) further improved discrimination to 0.73 (95% CI 0.71 to 0.74). Including the AST/ALT ratio in a simple age and gender-adjusted model (Model 1b) slightly improved discrimination to 0.69 (95% CI 0.67 to 0.71) from the age and gender adjusted model (Model 1a), although this increase was not significant. Including the AST/ALT ratio with Framingham risk factors (AUC c*-*statistic: 0.72, 95% CI 0.71 to 0.74, Model 2b) or QRISK2 risk factors (AUC c*-*statistic: 0.73, 95% CI 0.71 to 0.74, Model 3b) resulted in no change in discrimination from the standard Framingham or QRISK2 risk factor models (Models 2a and 3a). Figure 1 shows no incremental change in discrimination for Framingham and QRISK2 risk factors models with the addition of the AST/ALT ratio. The age and gender model serves as a baseline model for comparison.

**Figure 2:**
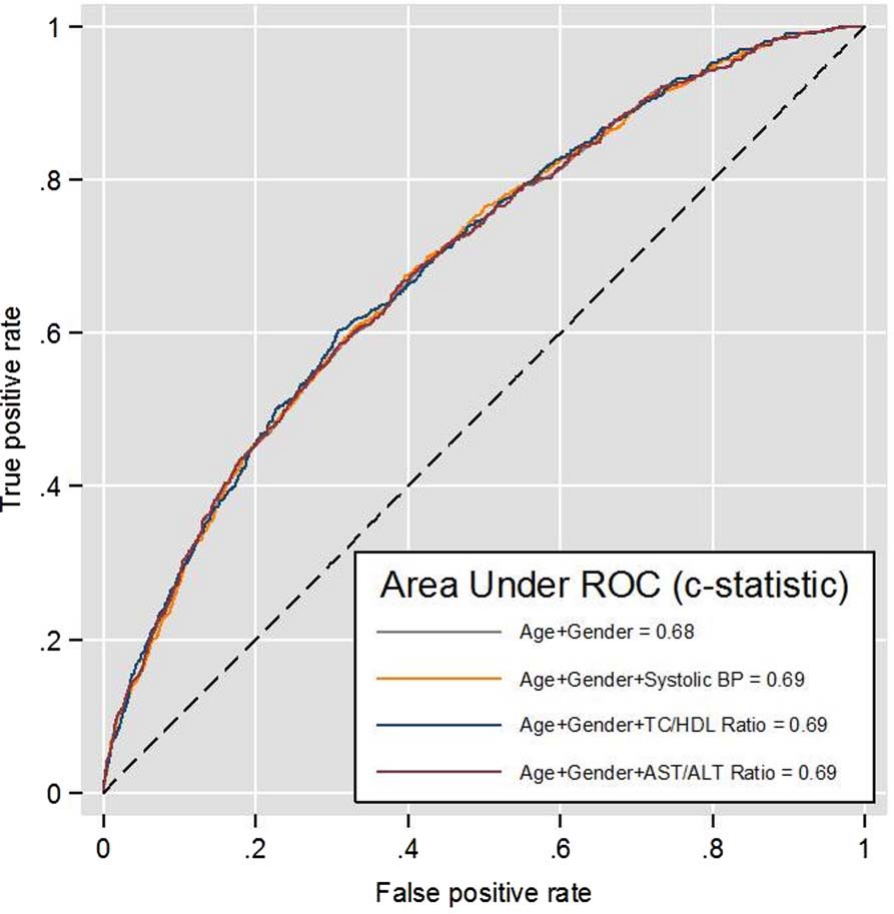
Receiver operating curves (ROC) of simple age and gender adjusted risk factor models for predicting 10-year risk of cardiovascular disease comparing the incremental benefits in discrimination from the AST/ALT ratio, systolic blood pressure, and the TC/HDL ratio. ALT, alanine transaminase; AST, aspartate transaminase; BP, blood pressure; HDL, high-density lipoprotein; TC, total cholesterol.

Two models were developed to assess the utility of the AST/ALT ratio compared against the standard biomarkers (systolic blood pressure and TC/HDL ratio). The comparator models of age and gender with either systolic blood pressure or the TC to HDL cholesterol ratio resulted in the same discrimination (AUC c*-*statistic: 0.69, 95% CI 0.67 to 0.71) as Model 1b including the AST/ALT ratio. Figure 2 shows that the discrimination increased by 1% with the inclusion of the AST/ALT ratio with age and gender, resulting in the same incremental benefit in discrimination as including systolic blood pressure or the TC/HDL ratio with age and gender.

### Sensitivity analysis

To take account of previous studies demonstrating the predictive value of the AST/ALT ratio for CVD, as previously reported in patients with NAFLD, is almost always associated with elevated ALT, a further sensitivity analysis was performed. To investigate the effects of this, we excluded patients with normal of low ALT using updated thresholds <19 U/L in women and <30 U/L in men.^24^ Discrimination of Framingham and QRISK2 CVD prediction models excluding these patients is shown in table 7. Compared to the primary analysis including all patients, this subgroup showed large improvements in predicting CVD when the AST/ALT ratio was included, resulting in a 5% and 4% increase in discrimination using the Framingham and QRISK2 risk factor models, respectively.

**Table 7:** Discrimination of the prediction models derived from multivariate hazard models excluding patients with normal or low ALT

Models	AUC c-statistic	SE*	95% CI
AST/ALT excluded			
Framingham risk factors	0.66	0.013	0.64 to 0.69
QRISK2 risk factors	0.68	0.014	0.65 to 0.71
AST/ALT ratio included			
Framingham risk factors+AST/ALT ratio	0.71	0.013	0.68 to 0.73
QRISK2 Risk factors+AST/ALT ratio	0.72	0.013	0.69 to 0.74

Higher area under receiver operating curve (AUC c-statistic) shows better model performance.

*SE estimated by jack-knife procedure.

ALT, alanine transaminase; AST, aspartate transaminase.

## Discussion

There was no improvement in discrimination after the AST/ALT ratio was included in multivariate risk prediction models for CVD in a heterogeneous primary care population. Despite this, we have established in this study that elevated levels of the AST/ALT ratio are independently associated with increased risk of developing CVD within 10 years in men but not women. Additionally, by excluding patients with normal or low ALT levels, we have shown that the AST/ALT ratio confers larger benefits in predicting CVD in individuals with elevated ALT levels. Currently, there is no clear biological mechanism but as well as a marker of CVD risk, AST/ALT ratio stratifies severity of liver disease, and this is independent of features of the metabolic syndrome.^25^ Moreover, emerging evidence in NAFLD suggests that liver fibrosis (with AST/ALT ratio as a proxy marker) stratifies future CVD risk rather than steatosis alone.^12^ ^14^ ^18^

The AST/ALT ratio being associated with CVD in men may be related to higher prevalence of liver disease in men. For instance, nearly 60% of incident cases of cirrhosis in the UK from 1998 to 2009 diagnosed in primary care occurred in men.^26^ Further, the risk of NAFLD increases with being obese or insulin resistant, which again are more common in men. As diabetes is an established risk factor for CVD in all established risk prediction tools while also being a primary risk factor of NAFLD,^27^ the effects of elevated AST/ALT ratio is likely masked by diabetes being present in the model. This was likely the case in this study as elevated AST/ALT ratios were also strongly and significantly associated with diabetes (OR=2.51, 95% CI 2.31 to 2.72). However, when we removed diabetes as well as other established risk factors, a simple age and gender adjusted model including AST/ALT ratio had a similar incremental improvement in predictive accuracy than other well-established CVD risk factors, such as, TC/HDL and systolic blood pressure. This implies that the AST/ALT ratio may have some clinical utility in CVD risk prediction, which is lost when diabetes is incorporated into the model.

## Clinical implications

Our analysis suggests that the AST/ALT ratio should not be included in current CVD risk prediction tools for the general primary care population. However, in the era of stratified medicine, those with raised AST/ALT ratio may represent a higher risk subgroup that could benefit from closer monitoring, particularly when ALT is raised. Latest UK guidelines^2^ ^28^ in primary care recommend clinicians to exclude type I diabetic patients in the use of CVD risk assessment tools as the calculated CVD risk may not be reliable, particularly in younger patients. As diabetes is a spectrum of disease which has been simplified as a binary variable in all standard CVD risk tools, a continuous variable such as the AST/ALT ratio may confer additional advantages for further stratification of these higher risk subgroups. Other potential risk factors for liver disease in subgroup populations with high obesity and alcohol usage should also warrant further analysis in which the AST/ALT ratio may confer larger benefits. Emerging evidence (including the findings in this study) showing the utility the AST/ALT ratio in stratifying liver disease and CVD risk in certain subgroups may inform future guideline development as stronger research evidence emerges. Given that the cost of either analyte is relatively cheap, the AST/ALT ratio's utility in predicting future CVD risk in groups such as those with elevated ALT, type II diabetes, and features of metabolic syndrome, alcohol usage or a combination of these factors may be extremely cost-effective. More broadly, the US Preventive Services Task Force,^29^ American Heart Association/American College of Cardiology^30^ and the recent Joint British Societies^28^ recommend now recommend revisiting the value of novel markers in risk prediction tools as more evidence becomes available.

In the context of identifying patients who should not be prescribed statin, the latest NICE lipid modification guidelines^2^ state that either AST or ALT should be assessed prior to starting statins. The implications of this are that mildly elevated levels will be wrongly seen as a contraindication to the initiation of statins. This study, however, reinforces the concept that liver transaminases, and specifically an elevated AST/ALT ratio, should be seen as identifying those with a greater need for a statin because of increasing CVD risk rather than a contraindication because of the relatively rare occurrence of a statin-induced liver injury.^31^ ^32^

## Limitations

Imputation of three QRISK2 risk factors: BMI, Townsend deprivation score and ethnicity were required to preserve the integrity of the sample size. However, our analysis, using only Framingham risk factors which contained complete data, suggests that imputation did not significantly alter the results as there was similar discrimination between the Framingham and QRISK2 risk factor models. Ethnicity, in the original QRISK2 algorithm, comprises eight UK ethnic groups, but was limited to two groups in this analysis. More specific ethnic group recording in primary care records was particularly poor. While multiple imputation has limitations, in particular when data are not ‘missing at random’, the approach has been recommended for epidemiological studies and recognised to have to statistically validity in the development of primary care risk prediction tools.^33^

In addition, our results from modelling Framingham risk factors for predicting 10-year CVD risk showed that TC was not significantly associated with CVD while the original published Framingham risk model^4^ shows a significant association with CVD. This discrepancy can be explained by the fact that the Framingham cohort is derived from a true prospective cohort of individuals with an inclusion criterion of untreated, fasting cholesterol measured at baseline. Although we excluded patients on statins at baseline, it is impossible to distinguish between fasting and non-fasting levels from primary care computer records. However, we did find that the TC/HDL ratio was a significant and better predictor of CVD than TC alone, which supports the QRISK2^3^ algorithm's use of the TC/HDL ratio instead of TC alone when developing risk algorithms from a primary care database.

Furthermore, other sources of AST include skeletal and cardiac muscle, with levels of AST increasing after acute MI. To account for this, we have excluded all patients at baseline with a history of CVD, including acute MI. Unless patients had undocumented MI in UK primary care records and secondary care linked records, which is highly unlikely given the significance of the event, this would not have likely been a confounding factor.

Finally, although the AST/ALT ratio is documented in a significant number of patients in this primary care database, there may be some ascertainment bias for measuring of these liver markers. Clinicians may have measured levels in patients who they suspect are at high risk of liver disease or as a marker for other confounding factors such as the decision to start and monitor statins and diabetes check-ups. However, the study cohort, comprising of a large sample of the UK general population, was not systematically different from the general population.
